# Supplementary material for: Anomaly Detection in Biological Early Warning Systems Using Unsupervised Machine Learning
Source: Sensors (Basel). 2023 Mar 1;23(5):2687. doi: 10.3390/s23052687 (PMC10007031; doi:10.3390/s23052687)
Supplement: Supplementary file 1 [file sensors-23-02687-s001.zip › sensors-2081746-supplementary.pdf]

Anomaly detection in biological early warning systems using unsupervised machine learning

Aleksandr N. Grekov [1,2], Aleksey A. Kabanov [2,*], Elena V. Vyshkvarkova [1], Valeriy V. Trusevich [1]

1 Institute of Natural and Technical Systems, Sevastopol 299011, Russia
2 Department of Informatics and Control in Technical Systems, Sevastopol State University, Sevastopol 299053, Russia
* Correspondence: kabanovaleksey@gmail.com

Table S1 – Example of raw data (valves opening value in mm)

date_time	M1	M2	M3	M4	M5	M6	M7	M8	M9	M10	M11	M12	M13	M14	M15	M16
2017-02-26 00:00:00	-6,8	7	4,43	6,85	5,5	7,45	2,96	1,25	3,91	8,92	4,43	4,07	4,28	3,95	7,09	7,65
2017-02-26 00:00:10	-6,8	7	4,44	6,85	5,48	7,45	2,96	1,25	3,91	8,92	4,44	4,06	4,28	3,95	7,09	7,65
2017-02-26 00:00:20	-6,8	7	4,43	6,85	5,49	7,45	2,96	1,25	3,91	8,92	4,43	4,07	4,28	3,95	7,09	7,67
2017-02-26 00:00:30	-6,81	6,99	4,43	6,86	5,5	7,45	2,96	1,25	3,91	8,92	4,44	4,07	4,29	3,95	7,09	7,64
2017-02-26 00:00:40	-6,8	7	4,44	6,85	5,49	7,46	2,96	1,25	3,91	8,92	4,43	4,07	4,29	3,95	7,09	7,66
2017-02-26 00:00:50	-6,8	7	4,43	6,85	5,49	7,45	2,96	1,25	3,91	8,92	4,44	4,07	4,27	3,95	7,1	7,66
2017-02-26 00:01:00	-6,8	7	4,44	6,85	5,48	7,45	2,96	1,25	3,9	8,92	4,43	4,07	4,28	3,95	7,09	7,66
2017-02-26 00:01:10	-6,81	7	4,44	6,86	5,48	7,45	2,96	1,25	3,91	8,93	4,44	4,07	4,28	3,95	7,09	7,66

Confusion matrices

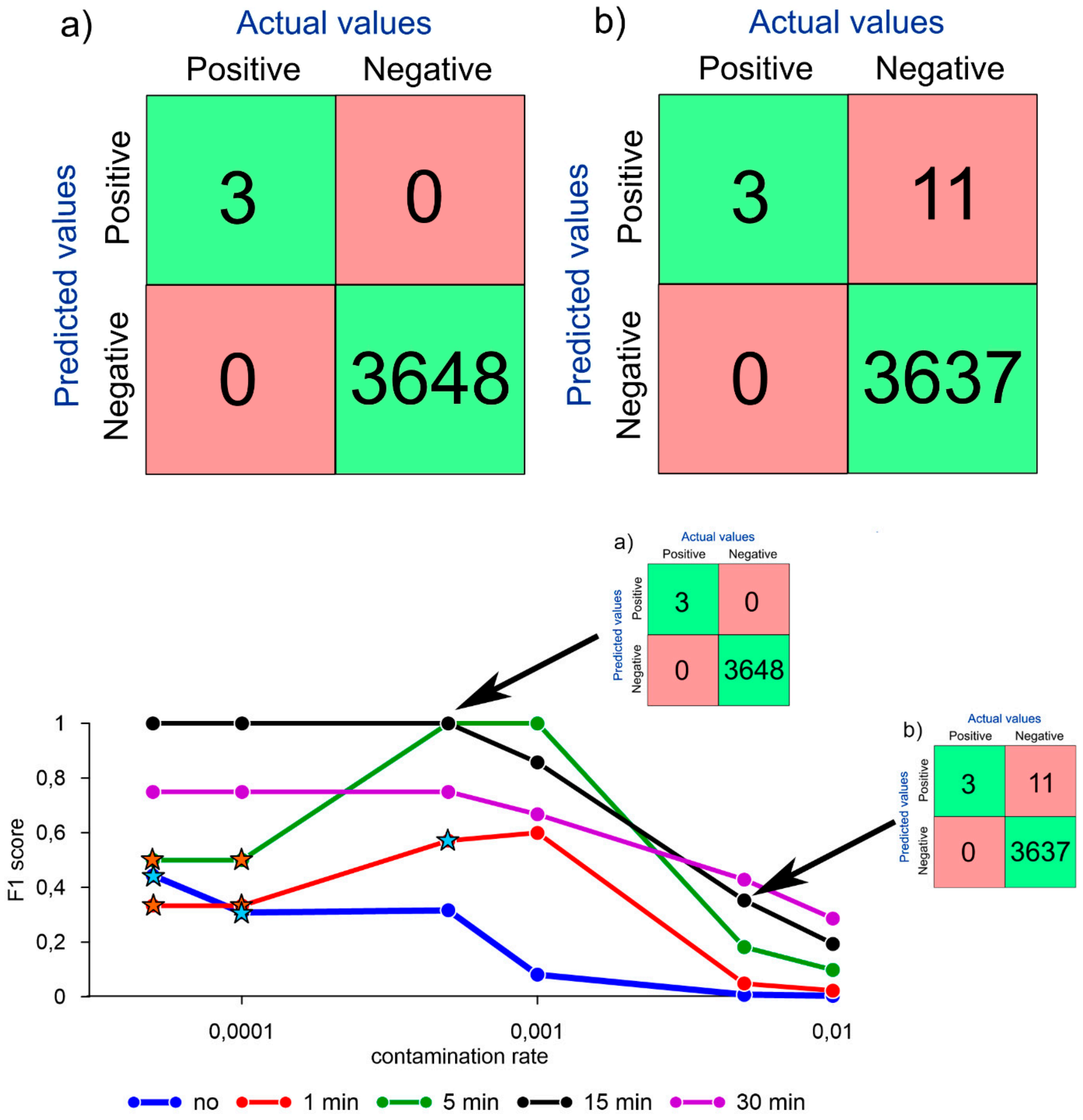

Figure S1. Confusion matrices for elliptic envelope algorithm models:
(a) 15 min average time, cr = 0.0005, F1 score = 1;
(b) 15 min average time, cr = 0.005, F1 score = 0.353

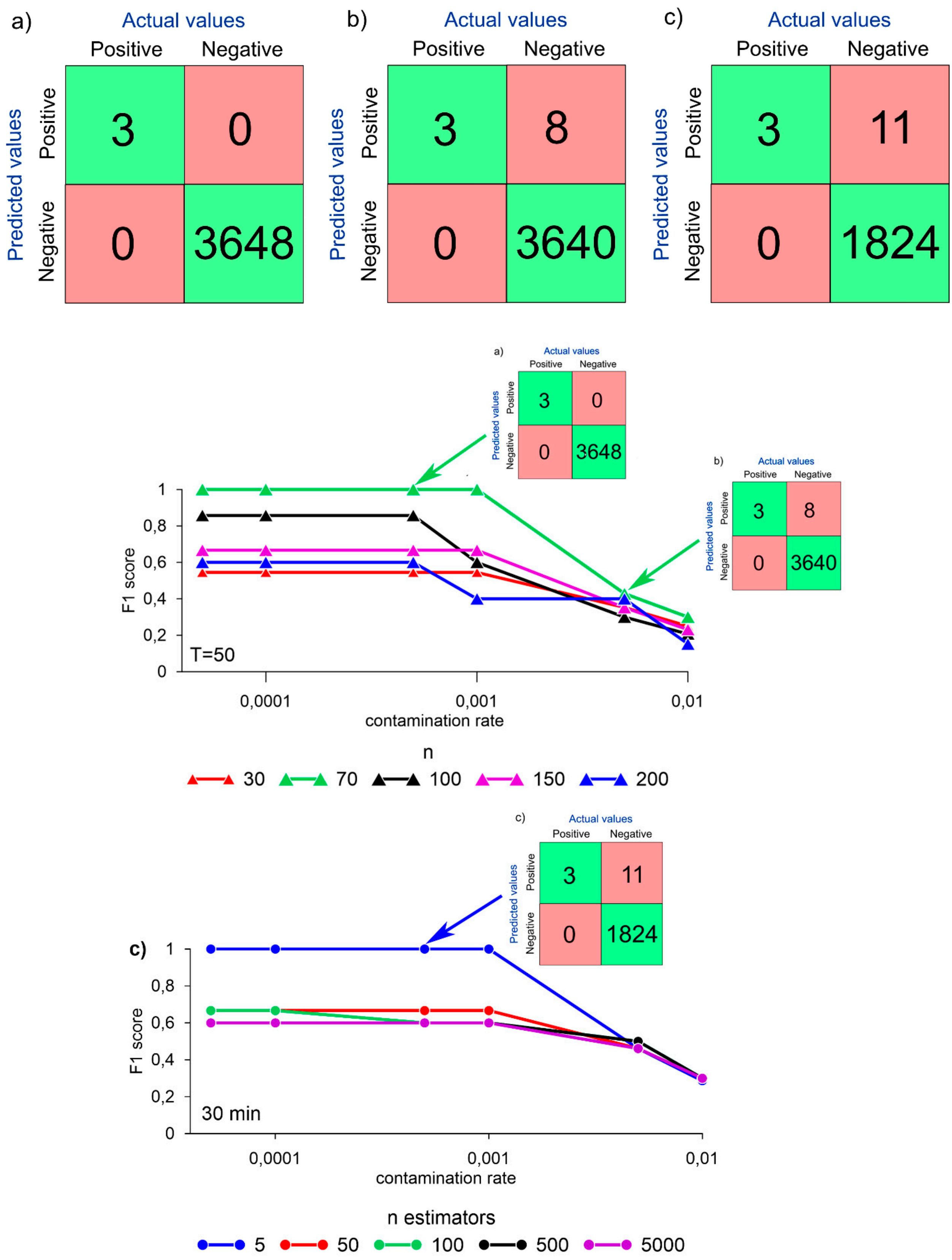

Figure S2. Confusion matrices for iForest algorithm models:
(a) 15 min average time, cr = 0.0005, max_samples = 70, n=50, F1 score = 1;
(b) 15 min average time, cr = 0.005, max_samples = 70, n=50, F1 score = 0.429;
(c) 30 min average time, cr = 0.0005, max_samples = 256, n=5, F1 score = 1

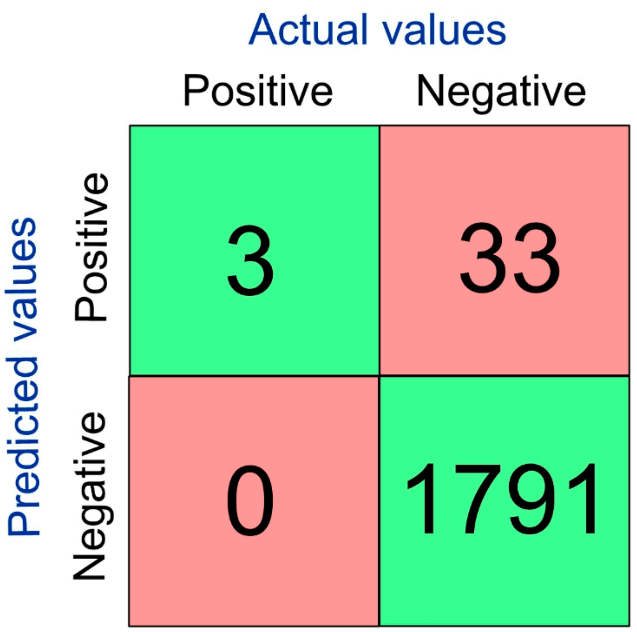

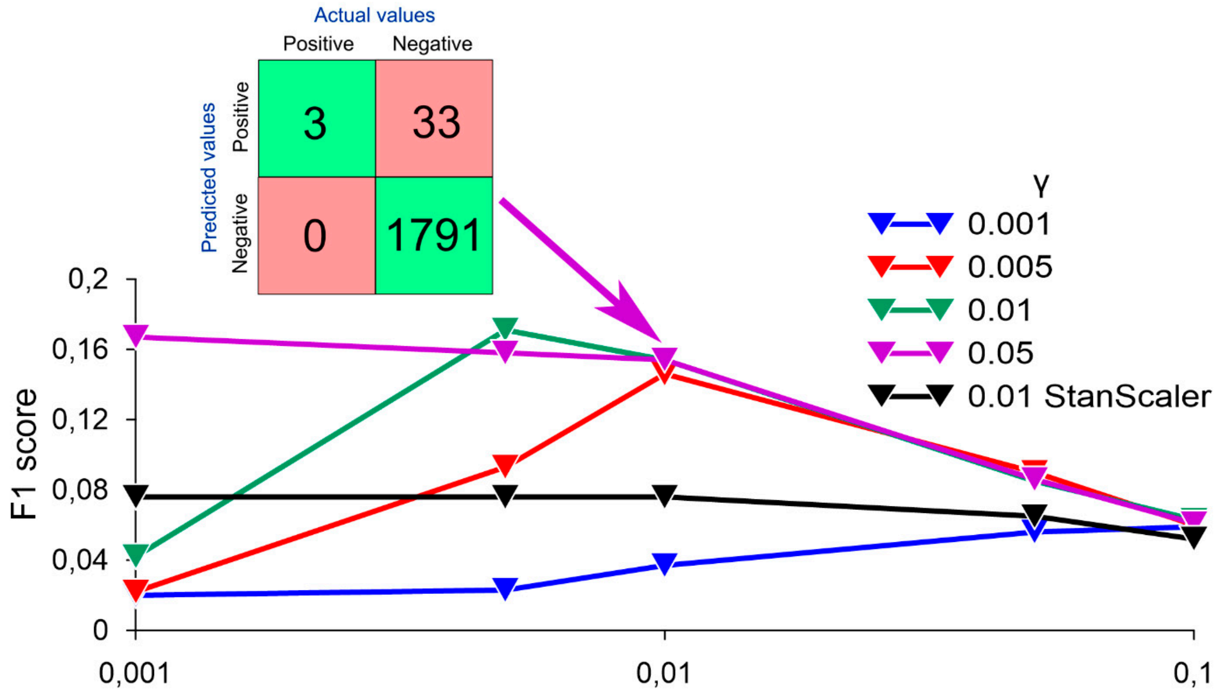

Figure S3. Confusion matrix for One-Class SVM algorithm model: 30 min average time, RBF, MinMaxScaler, cr = 0.01, γ =0,05, F1 score = 0,154;

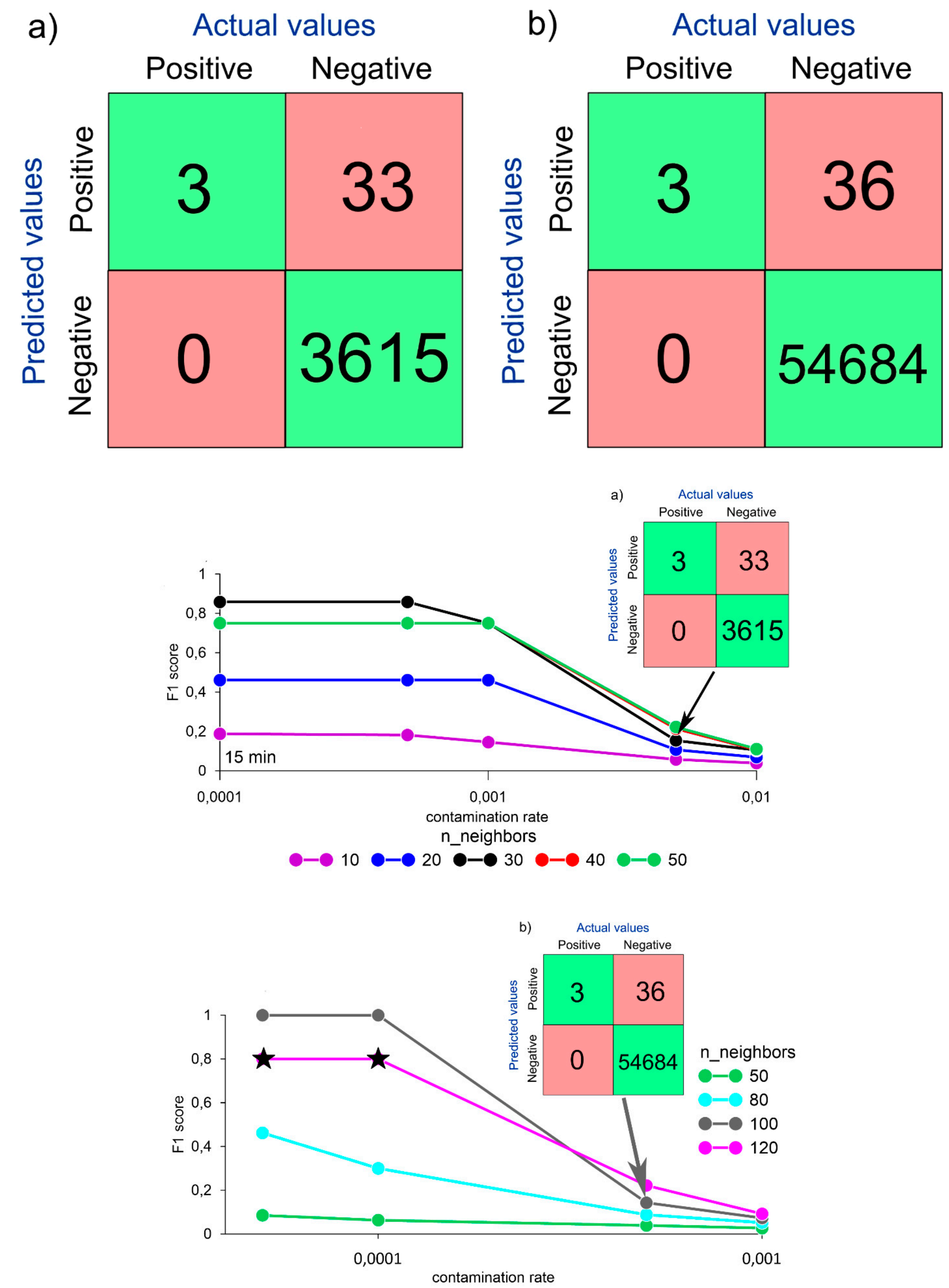

Figure S4. Confusion matrices for LOF algorithm models:
(a) 15 min average time, cr = 0.005, k-neighbors =30, F1 score = 0.154;
(b) 1 min average time, cr = 0.0005, k-neighbors =100, F1 score = 0.143;
